# Preoperative chemoradiotherapy for locally advanced gastric cancer

**DOI:** 10.1186/1748-717X-8-6

**Published:** 2013-01-04

**Authors:** Joseph M Pepek, Junzo P Chino, Christopher G Willett, Manisha Palta, Dan G Blazer III, Douglas S Tyler, Hope E Uronis, Brian G Czito

**Affiliations:** 1 Department of Radiation Oncology, Duke University School of Medicine, Box 3085, Durham, NC, 27710, USA; 2 Department of Surgery, Duke University School of Medicine, Durham, NC, USA; 3 Department of Medicine, Division of Medical Oncology, Duke University School of Medicine, Durham, NC, USA

**Keywords:** Gastric cancer, Chemoradiotherapy, Neoadjuvant

## Abstract

**Background:**

To examine toxicity and outcomes for patients treated with preoperative chemoradiotherapy (CRT) for gastric cancer.

**Methods:**

Patients with gastroesophageal (GE) junction (Siewert type II and III) or gastric adenocarcinoma who underwent neoadjuvant CRT followed by planned surgical resection at Duke University between 1987 and 2009 were reviewed. Overall survival (OS), local control (LC) and disease-free survival (DFS) were estimated using the Kaplan-Meier method. Toxicity was graded according to the Common Toxicity Criteria for Adverse Events version 4.0.

**Results:**

Forty-eight patients were included. Most (73%) had proximal (GE junction, cardia and fundus) tumors. Median radiation therapy dose was 45 Gy. All patients received concurrent chemotherapy. Thirty-six patients (75%) underwent surgery. Pathologic complete response and R0 resection rates were 19% and 86%, respectively. Thirty-day surgical mortality was 6%. At 42 months median follow-up, 3-year actuarial OS was 40%. For patients undergoing surgery, 3-year OS, LC and DFS were 50%, 73% and 41%, respectively.

**Conclusions:**

Preoperative CRT for gastric cancer is well tolerated with acceptable rates of perioperative morbidity and mortality. In this patient cohort with primarily advanced disease, OS, LC and DFS rates in resected patients are comparable to similarly staged, adjuvantly treated patients in randomized trials. Further study comparing neoadjuvant CRT to standard treatment approaches for gastric cancer is indicated.

## Introduction

Postoperative chemoradiotherapy (CRT), perioperative chemotherapy (ChT) or postoperative ChT are the current standards of care for resectable gastric cancer based on the outcomes of phase III randomized trials [1-4]. While these strategies have been shown to improve disease-related outcomes compared to surgery alone, they are associated with higher rates of treatment-related morbidity. Illustrating this fact, only 64% of patients in the Intergroup-0116 trial and 42% in the Medical Research Council Adjuvant Gastric Infusional Chemotherapy (MAGIC) trial were able to complete their prescribed treatment courses [1,2].

Preoperative CRT has been established as the primary treatment modality in other gastrointestinal malignancies, including esophageal [5-8] and rectal cancer [9]. Preoperative CRT has several potential biological and technical advantages. The presence of intact tumor vasculature and oxygenation may enhance responsiveness to radiotherapy (RT) and systemic therapy. This treatment approach may also sterilize the surgical field, potentially reducing the risk of local tumor dissemination at resection. Preoperative CRT may also allow design of smaller and more accurate radiation treatment fields which could improve treatment tolerance, as well as identify patients with biologically aggressive disease in whom surgery should be avoided. In addition, postoperative morbidity following upfront gastrectomy can be significant, resulting in the delay or omittance of adjuvant therapy administration [1,2,10]. Lastly, preoperative CRT may result in tumor downstaging, facilitating curative (R0) resection rates while decreasing the risk of local tumor recurrence.

There are scant prospective data on clinical outcomes utilizing preoperative RT for gastric cancer. Trials of neoadjuvant RT or CRT compared to surgery alone have shown an overall survival (OS) advantage with preoperative treatment in patients with esophageal and gastric cardia adenocarcinomas [5,11]. Additionally, several small phase II trials examining the role of induction ChT followed by preoperative CRT for gastric cancer showed encouraging rates of pathologic complete response (pCR) and R0 resections, with acceptable rates of acute and late toxicity [12-14]. Single institution experiences examining a neoadjuvant approach for gastric cancer have also been published [15-17], but data looking at preoperative CRT without induction ChT are limited. The intent of this study is to examine our institutional experience with neoadjuvant CRT for potentially resectable gastric adenocarcinoma, evaluating treatment-related toxicity, R0 resection rates, pCR rates, and disease-related endpoints.

## Methods

This Institutional Review Board-approved study included patients who underwent preoperative CRT for non-metastatic gastroesophageal (GE) junction and gastric adenocarcinoma at Duke University Medical Center between 1987 and 2009. Medical records, pertinent radiographs and RT fields were reviewed to obtain patient demographics, operative findings, pathology, toxicity, and to determine local-regional and distant recurrence patterns. Tumor epicenter, GE junction involvement and tumor (T) and nodal (N) staging were determined using upper endoscopy, radiographic imaging and/or endoscopic ultrasound (EUS).

The institutional policy has been to proceed directly to resection for early stage gastric cancer in most patients. In general, patients with locally advanced disease, which includes those with large primary tumors (≥ T3 tumors), extensive local-regional nodal involvement or where a R0 surgical resection may not be feasible initially, are usually referred for preoperative CRT following evaluation by a multidisciplinary team.

Only Siewert type II/III GE junction and gastric adenocarcinomas were included in this analysis [18]. Patients with direct tumor extension into adjacent organs who were still thought to be candidates for resection were included. Patients were excluded if the initial treatment plan was for definitive or palliative CRT, or if they were found to have metastatic disease during pre-treatment workup.

Guidelines for preoperative RT planning for gastric cancer have been previously published and can serve as a framework for our general approach to treatment field design [19]. External beam RT was delivered via high energy linear accelerators using 6 MV or 15 MV photons. Multi-field external beam RT plans were typically designed to cover the primary tumor and local-regional lymph node basins. In recent years, a deep inspiratory breath-hold technique during RT was utilized for patients with significant tumor motion. Induction, concurrent and adjuvant ChT selection was at the discretion of the treating Medical Oncologist. Surgical resection typically occurred 4–6 weeks after CRT completion. The type of surgery depended upon the treating surgeon’s preference, primary tumor location and disease extent.

Each patient was staged using the American Joint Committee on Cancer gastric cancer staging guidelines, 6th edition. Tumor downstaging was defined as a decrease in either T stage and/or N stage at the time of resection. Patterns of failure were determined by imaging studies and/or by findings obtained from diagnostic procedures such as laparoscopy, laparotomy or computed tomography (CT)-guided biopsies. Local recurrence was defined as disease recurrence within the remaining stomach, the operative bed or local-regional (lower paraesophageal, gastrohepatic, celiac, superior mesenteric, porta hepatis, and splenic) lymph nodes basins. Radiographic lymph node recurrences were defined as lymph nodes ≥ 1 cm on CT which had increased in size on serial imaging and/or were hypermetabolic on positron emission tomography (PET). Distant recurrence was defined as any recurrence outside the aforementioned sites. A consensus opinion was reached by two authors (J.P. and B.C.) on all suspected cases of local and distant recurrences as a means to improve accuracy.

The Kaplan-Meier method was used to estimate 3-year OS, disease-free survival (DFS) and local control (LC) probabilities with 95% confidence intervals (95% CI). All disease-related endpoints were calculated from the preoperative therapy start date. Testing for time to event differences between groups was performed using the log-rank (Mantel-Cox) test. Analysis was performed using SPSS 19.0.0 (IBM Corporation, Somers, NY).

Toxicity was assigned using the National Cancer Institute Common Toxicity Criteria for Adverse Events version 4.0 (CTCAE v4.0). Acute toxicity was defined as any toxicity from the start of preoperative therapy until surgery. Late toxicity was defined as any toxicity greater than 90 days after surgery.

## Results

Forty-eight patients were included in the present analysis. Thirty-three were treated from the year 2000 onward. Median follow-up was 42 months (range 17–144). Twenty-nine patients (61%) were clinical stage III, and five patients (10%) were clinical T4 at presentation. Thirty-one (65%) were clinically node positive. Staging PET scan was used for 19 patients (40%) and CT scan was used for 28 (59%). One patient with stage IB disease did not receive radiographic staging due to pregnancy at the time of diagnosis. Thirty-six patients (75%) underwent baseline EUS prior to CRT. Fourteen patients underwent pre-treatment laparoscopy or laparotomy.

Thirty-five patients (73%) had proximal tumors, which include GE junction, cardia, and fundus tumors. Eight patients (17%) had distal tumors, which include lesions in the antrum and pylorus. Five patients (10%) had linitis plastica. Most tumors (n = 25) had poorly differentiated histology. Patient and tumor characteristics are reported in Table 1.

**Table 1:** Patient and tumor characteristics for patients undergoing preoperative chemoradiotherapy for gastric adenocarcinoma (n = 48)

**Characteristic**	**No.**	**Value**	**%**
Age, years			
Median		60	
Range		28-79	
Gender			
Male	38		79
Female	10		21
Race			
Caucasian	35		73
African-American	11		23
Asian	2		4
Primary site location*			
Proximal	35		73
Distal	8		17
Linitis plastica	5		10
Stage at presentation (AJCC 6)			
IB	1		2
II	9		19
IIIA	26		54
IIIB	3		6
IV	4		8
Unknown T/N	5		10
Histologic differentiation			
Well	2		4
Moderate	15		31
Poor†	25		52
Not specified	6		13

*Proximal tumors involve lesions of the GE junction, cardia or fundus. Distal tumors include lesions of the antrum and pylorus; †Includes signet ring 6 (n = 17) and mucinous (n = 1) histology.

Median RT dose was 45 Gy (range 21.6-50.4). All patients received concurrent ChT and 40 patients (83%) received fluoropyrimidine-based ChT (Table 2). Four patients received one cycle of induction fluoropyrimidine-based ChT prior to starting RT. Six patients (13%) required a treatment break for a median of 4 days (range 2–23). Two patients (4%) were unable to complete the prescribed CRT treatment course; one patient experienced a gastric perforation during CRT and the other developed febrile neutropenia and failure to thrive toward the end of his CRT course.

**Table 2:** Treatment characteristics for patients undergoing preoperative chemoradiotherapy for gastric adenocarcinoma (n = 48)

**Characteristic**	**No.**	**%**
Concurrent chemotherapy		
5-FU or Capecitabine	19	40
5-FU/Carboplatin/Paclitaxel	11	23
5-FU/Cisplatin	6	13
5-FU/Mitomycin C	3	6
5-FU/Methotrexate	1	2
Cisplatin/Paclitaxel	6	13
Carboplatin/Paclitaxel	2	4
Surgical procedure		
Total gastrectomy	13	27
Partial gastrectomy	9	19
Esophagogastrectomy		
Transhiatal	5	10
Ivor-Lewis	4	8
Sweet	3	6
McKeown	1	2
Not specified	1	2
No surgical resection	12	25

Thirty-six patients (75%) underwent surgery. Total gastrectomy was performed in 13 patients, partial gastrectomy in 9 patients and esophagogastrectomy in 14 patients (Table 2). The median number of lymph nodes sampled was 6 (range 0–34). The R0 resection rate was 86% (n = 31). Overall tumor downstaging was seen in 18 of 28 patients (64%) who had EUS prior to starting preoperative therapy. T stage downstaging was seen in 19 patients (68%). Of the 36 patients who underwent surgery, 13 of 22 (59%) who were clinically node positive on initial evaluation were pathologically node negative. A pCR was seen in 7 of 36 patients (19%). Nine (25%) received adjuvant ChT after a R0 resection. Of those, seven patients received postoperative fluoropyrimidine-based ChT. Patients did not undergo definitive surgical resection due to distant metastases found on restaging scans or exploratory laparotomy (n = 9), patient refusal (n = 2) or poor performance status following preoperative CRT (n = 1). One patient who underwent gastrectomy was found to have peritoneal carcinomatosis on final pathology despite negative intraoperative frozen sections.

**Figure 1:**
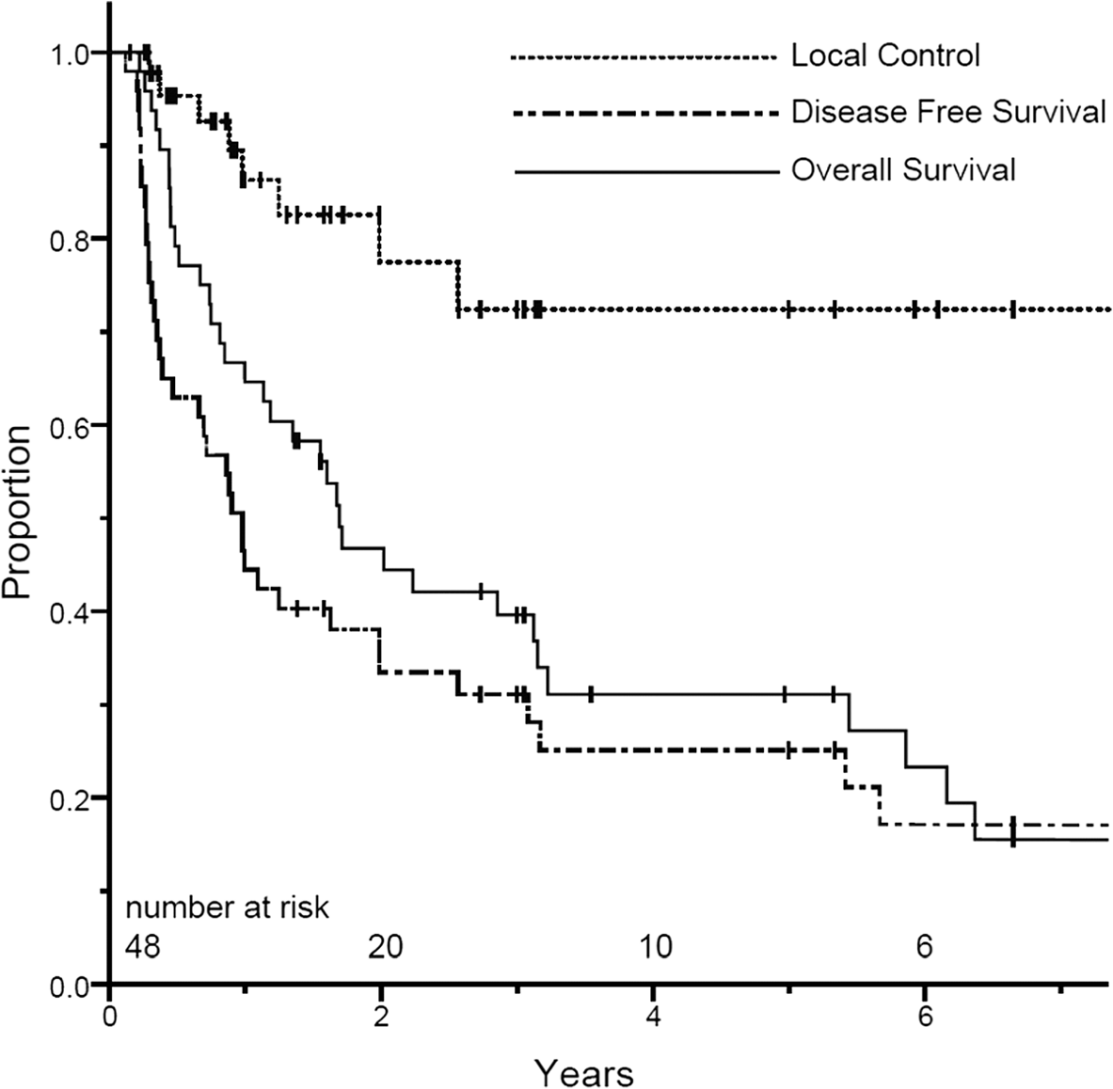
Kaplan-Meier estimates for overall survival, disease-free survival and local control, all patients.

For the entire cohort, the first site of failure was local-regional only in 3 patients, synchronous local-regional and distant in 5 patients and distant only in 19 patients. One local recurrence occurred in a patient who refused surgery after completing CRT and developed a gastric recurrence 26 months later. The most common sites of distant recurrence were the peritoneal cavity (n = 10) and liver (n = 8). Three-year actuarial LC and DFS rates for the entire cohort at 3 years were 72% (95% CI 55–90) and 30% (95% CI 17–44), respectively. Median OS was 20 months (range 3–146) and the 3-year OS for the entire cohort was 40% (95% CI 25–54) (Figure 1).

**Figure 2:**
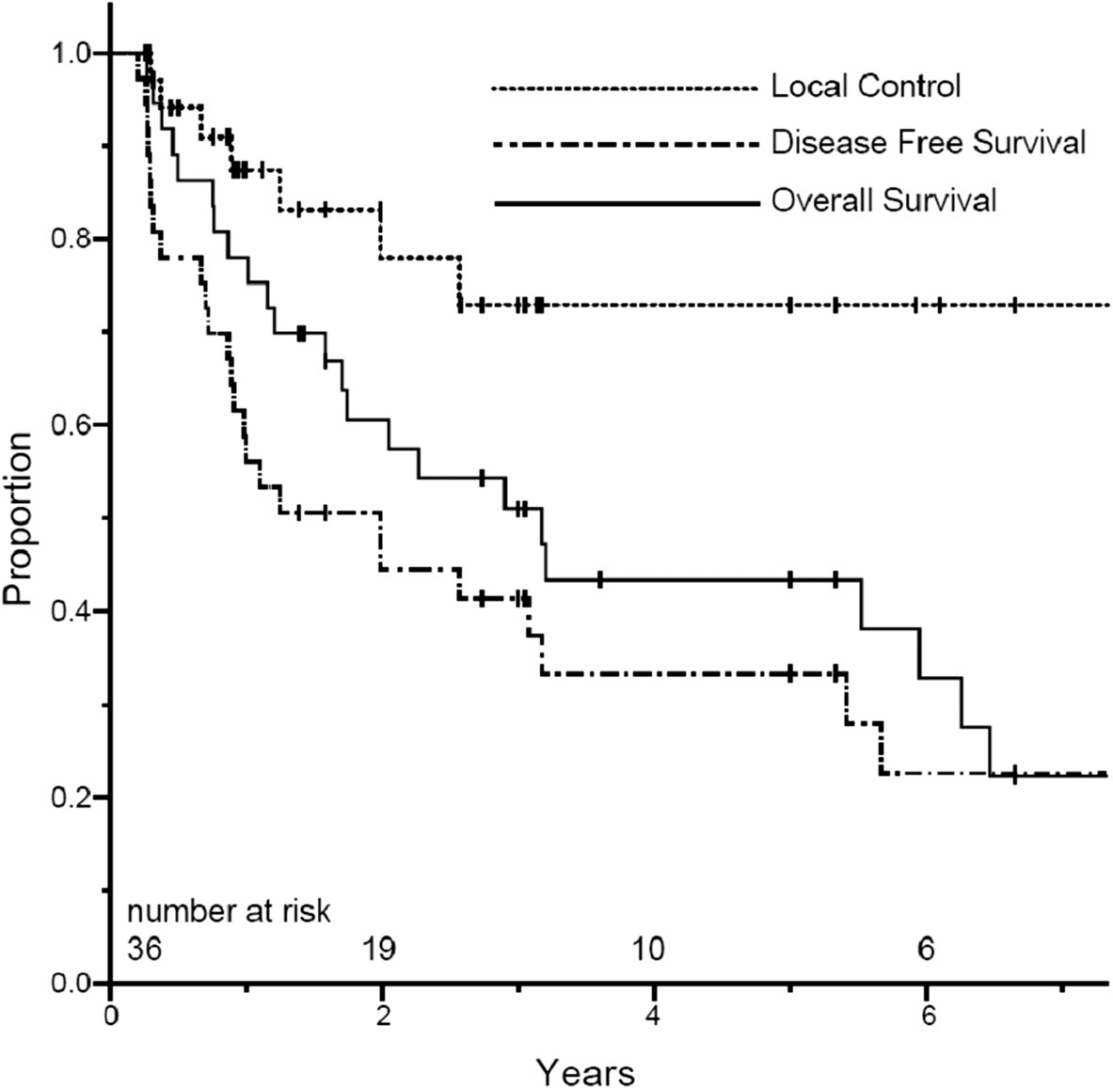
Kaplan-Meier estimates for overall survival, disease-free survival and local control for patients undergoing surgery.

For the 36 patients who underwent surgery, the first site of failure was local-regional only in 2 patients, synchronous local-regional and distant in 5 patients, and distant only in 10 patients. For the 7 patients who achieved pCR, there were no local recurrences and three distant recurrences (liver (n = 2) and brain (n = 1)). Three-year actuarial LC and DFS rates for patients who underwent surgery were 73% (95% CI 55–91) and 41% (95% CI 24–57), respectively. The 3-year OS rate for patients who underwent surgery was 50% (95% CI 33–68) (Figure 2). Median OS for patients who completed combined modality therapy followed by surgical resection was 37 months. Those who completed CRT but did not undergo surgery had a median OS of 6 months. There was no difference in OS at 3 years for patients who achieved a pCR versus those who did not (57% vs. 43%, p = 0.60). However, in patients achieving R0 resection (compared to R1 resection), there were statistically significant improvements at 3 years in OS (54% vs. 0%, p = .045) and DFS (42% vs. 0%, p = 0.002).

Acute grade 3–4 CRT-related toxicity is listed in Table 3. Two patients (6%) died within 30 days following surgery. One died due to gastrointestinal hemorrhage and the other died from postoperative sepsis. Four patients (11%) experienced a postoperative anastomotic leak that required intervention; an additional 3 patients (8%) had asymptomatic anastomotic leaks which were managed conservatively. Table 4 lists toxicities at least 3 months following surgery. All late grade 3 gastrointestinal toxicity was due to prolonged feeding tube dependence stemming from GI dysmotility (n = 4) or dysphagia requiring esophageal dilation (n = 9). The actuarial rate of chronic (≥ 3 months) dysphagia requiring esophageal dilation was 21% at 1 year (median time to dilation 5.7 months). There was one late grade 5 event stemming from complications following gastric perforation occurring during CRT.

**Table 3:** Grade 3–4 chemoradiotherapy-associated acute toxicity (n = 48)

	**Toxicity**	**Grade 3-4**	
		**n**	**%**
**Hematologic Toxicity**	Worst Hematologic Toxicity	18	38
	Anemia	1	2
	Leukopenia	15	31
	Thrombocytopenia	3	6
**Non-Hematologic Toxicity**	Worst Non-Hematologic Toxicity	5	10
	Nausea	3	6
	Dehydration	3	6
	Diarrhea	0	0
	Dysphagia	1	2
	Perforation	1	2

**Table 4:** Long-term (≥ 3 month) toxicity for patients undergoing surgery (n = 36)

**Toxicity**	**Grade 2**	**Grade 3**	**Grade 4**	**Grade 5**
**Worst Overall**	3 (8%)	12 (33%)	0	1 (2%)
**Stomach/Bowel**	3 (8%)	4 (11%)	0	1 (2%)
**Esophagus**	1 (3%)	9 (25%)	0	0
**Musculoskeletal**	1 (3%)	0	0	0
**Other**	1 (3%)	0	0	0

Percentages are in parentheses.

## Discussion

The primary treatment modality for patients with localized gastric cancer is surgery. However, rates of both local and distant recurrence remain high following curative intent resection alone [20-23]. Several small prospective studies have examined the use of induction ChT prior to preoperative CRT for potentially resectable gastric cancer [12-14]. Single institution experiences using either preoperative ChT and/or CRT have also been described [15-17], although the data examining a neoadjuvant approach in gastric cancer remains limited. Our institution has favored an approach of neoadjuvant CRT without induction ChT for patients primarily with locally advanced disease, prompting the present analysis of treatment-related toxicities and outcomes for this group of patients.

Preoperative CRT was well tolerated in our series, with 96% of patients able to complete the prescribed treatment course and only six patients requiring treatment break. Similarly, in a pilot study from Lowy et al., 96% (23 of 24 patients) were able to complete neoadjuvant fluoropyrimidine-based CRT without treatment break [24]. These studies are in contrast to results from the Intergroup-0116 trial where only 64% of 281 patients were able to complete the prescribed postoperative protocol [1]. Improved compliance and acceptable acute toxicity rates with neoadjuvant CRT seen in our series may be due in part to smaller RT treatment fields and/or improved performance status at the time of CRT compared to the post-gastrectomy setting.

Acute and late toxicity data assessing neoadjuvant CRT for gastric cancer are limited [12,13,24]. In our series, four surgical patients (11%) experienced symptomatic anastomotic leak requiring intervention, which is similar to rates seen in a pilot study of preoperative CRT [24] and a Dutch Gastric Cancer Group trial evaluating extended lymphadenectomy [25] for gastric cancer. With respect to late toxicity, 5% of patients in RTOG 9904 evaluating preoperative CRT experienced late grade 3 toxicity [12], which is lower than in our series. Most occurrences of late grade 3 toxicities in our cohort were dysphagia, with an estimated 21% actuarial risk of requiring esophageal dilation at one year. It is possible that these comparative differences may be attributable to variations in scoring toxicity between studies.

Thirty-day postoperative mortality was 6% in our analysis, which is consistent with previously reported mortality rates examining neoadjuvant CRT for gastric [14,24] and esophageal malignancies [5,8,26]. The influence of preoperative CRT on perioperative mortality remains debatable when compared to upfront surgical resection. In the POET trial by Stahl et al., preoperative CRT was associated with a non-significant increase in postoperative mortality compared to a ChT alone approach [27]. However, the CROSS trial showed no difference in operative mortality for patients who received preoperative CRT versus those undergoing surgery alone for esophageal cancer [8]. Similarly, previously published data from our institution suggested that induction therapy for esophagogastrectomy patients was not associated with an increased risk of surgical mortality on multivariable analysis [28].

Historically, the inability to achieve a curative resection with surgery alone for gastric cancer is high and portends a poor prognosis [29]. One randomized trial demonstrated an improvement in OS for gastric cancer patients achieving R0 resection [30]. Additionally, the MAGIC trial showed a statistically significant improvement in R0 resections among surgical patients who received preoperative ChT [2]. Using these trials as guides, it can be inferred that a preoperative approach may improve disease-related outcomes. The R0 resection rate for those undergoing surgery in our series (86%) is comparable to prospective studies utilizing neoadjuvant CRT for gastric cancer [12-14,24]. This is particularly encouraging as many patients had locally advanced, potentially unresectable disease at presentation with accompanying adverse histologic features, including signet ring cell histology and linitis plastica [31]. Our data underscore the importance of achieving a R0 resection, with 3-year OS of 54% compared to 0% for those undergoing R1 resection.

Several series have demonstrated that pathologic response to CRT is predictive of patient outcomes [12,13,15-17]. Three phase II trials from Ajani et al. showed pCR rates ranging from 20% to 30% with the use of induction CT followed by concurrent CRT, [12-14] with associated improvements in OS and DFS in these patients. A series from M.D. Anderson reported a 23% pCR rate with either induction ChT followed by concurrent CRT or concurrent CRT for both GE junction and gastric malignancies. In this study, pCR was the only statistically significant predictor of LC based on multivariable analysis [15]. Similarly, another recent report showed lower recurrence rates and greater OS, DFS and relapse-free survival in the pCR group versus the non-pCR group [16]. The pCR rate in the present analysis appears consistent with other series, but did not translate into improved OS in our cohort. Although LC was 100% in the pCR group, the low absolute numbers of patients and high distant failure rates potentially diluted any impact of pCR within our study. Nevertheless, most data suggest that gastric cancer patients who have a pCR have more favorable outcomes. Data examining preoperative ChT alone for resectable gastric cancer show pCR rates of only 0-7% [2,32,33], suggesting that the addition of RT with concurrent ChT may improve tumor response and disease-related endpoints compared to a ChT alone approach. This was demonstrated in a recent randomized trial evaluating preoperative ChT versus CRT in GE junction cancer patients [27].

While the number of events are small, our data suggest that both local and distant recurrences are still problematic despite aggressive combined modality therapy. Most patients recurred distantly as their first site of recurrence, which is concordant with other published series [12,16]. The actuarial local recurrence rate in our cohort was 28% for all patients and 27% in the subset of patients who underwent gastrectomy. While these rates are slightly higher than expected, even for those with a R0 resection, this may be attributable to the aggressive and locally advanced nature of disease seen in our cohort as well as variability in RT treatment field design over the study period. Optimizing ChT regimens, including the use of novel conventional and targeted biologic therapies, may be a potential way to improve pathologic response and R0 resection rates, reduce distant recurrences, and improve survival rates in future trials examining preoperative CRT. Advancements in pre-treatment staging are also critical to better select appropriate surgical candidates. Two such examples include PET/CT, which may improve accuracy for preoperative staging [34], and staging laparoscopy, which may identify the approximately 20-40% of patients with localized disease based on preoperative CT but who demonstrate peritoneal metastases at laparoscopy [35,36].

The present study has a number of limitations inherent to retrospective series. First, our cohort is limited by small patient numbers, resulting in few total events and wide confidence intervals for actuarial data. Second, clinical and pathological data were not collected prospectively which allows for selection bias to be introduced. Furthermore, local and distant recurrences were not always pathologically confirmed, with most diagnosed by CT imaging, potentially underestimating local and distant recurrences within the study. During surgery, a median of 6 lymph nodes were sampled which may be insufficient for adequate dissection of regional lymph nodes. However, the limited extent of lymph node dissection appears consistent with national data from the Surveillance, Epidemiology, and End Results (SEER) cancer registry which showed that a median of 8 lymph nodes were sampled during gastric cancer resection, with only 25% of patients having 15 or more lymph nodes examined [37]. Additionally, the impact of preoperative CRT on lymph node harvest in gastric cancer is not well defined. Nevertheless, a more extensive dissection may have improved disease-specific outcomes in our cohort given the potential for understaging with a limited lymph node sampling [37,38].

Despite these limitations, it is important to emphasize that many of these patients were referred for initial CRT due to concern for unresectability. This may result in a negative selection bias for patients who are referred for RT in this series, potentially leading to worse treatment-related outcomes. Even with these aggressive malignancies, R0 resection rates, pCR and survival outcomes are comparable to prospective series utilizing neoadjuvant CRT for gastric cancer (Table 5). Importantly, patients with rapidly progressive disease or with occult metastatic disease at presentation were spared the morbidity associated with surgical resection with this treatment approach. In our study, 25% of patients did not undergo gastrectomy due to the reasons stated above. Thus, we contend that neoadjuvant CRT can be a feasible and effective selection tool to identify patients with locally advanced disease who could proceed to surgery.

**Table 5:** Comparison of present series to prospective preoperative chemoradiotherapy trials in gastric cancer

**Series**	**Patients (n)**	R0 resection (%)*	pCR rate (%)*	Overall survival (%)**
M.D. Anderson (Lowy et al.)	24	95	11	N/A
Multi-institutional (Ajani et al.)	33	82	36	54 (2)
M.D. Anderson(Ajani et al.)	41	80	20	N/A
RTOG 9904 (Ajani et al.)	43	75	31	72 (1)
Current series (Pepek et al.)	48	86	19	40 (3)

* Percentages are based on number of patients who underwent surgery after preoperative chemoradiotherapy.

** Numbers in parentheses are actuarial data in years.

In conclusion, preoperative CRT for gastric cancer is reasonably well tolerated with acceptable rates of perioperative morbidity and mortality. R0 resection rates and pCR are encouraging with this treatment strategy. Despite evaluating a cohort with primarily advanced disease and adverse histologic features, LC, DFS and OS rates in resected patients are comparable to similarly staged, adjuvantly treated patients in randomized trials. Further study comparing neoadjuvant CRT to standard treatment approaches for gastric cancer is warranted.

Presented at the 52th Annual Meeting of the American Society of Radiation Oncology, San Diego, California, October 31-November 4, 2010.

## Competing interest

The authors declare that they have no competing interests.

## Authors’ contributions

JP and BC performed study conception and design. JP, JC and BC performed data collection, analysis and interpretation. JP, BC, MP, CW and DT assisted in manuscript writing. All authors read and approved the final manuscript.

## Synopsis

This single-institutional series evaluates the role of preoperative chemoradiotherapy in patients with locally advanced gastric cancer. Disease-related outcomes for resected patients appear comparable to similarly staged, adjuvantly treated patients in randomized trials with acceptable rates of perioperative morbidity and mortality.
